# Electrospun Donor/Acceptor Nanofibers for Efficient Photocatalytic Hydrogen Evolution

**DOI:** 10.3390/nano12091535

**Published:** 2022-05-02

**Authors:** Xiaoyu Lin, Yuanying Liang, Zhicheng Hu, Xi Zhang, Youcai Liang, Zhengwei Hu, Fei Huang, Yong Cao

**Affiliations:** State Key Laboratory of Luminescent Materials and Devices, Institute of Polymer Optoelectronic Materials and Devices, South China University of Technology, Guangzhou 510640, China; 201920117734@mail.scut.edu.cn (X.L.); liangyuanying1@hotmail.com (Y.L.); 201710102620@mail.scut.edu.cn (X.Z.); msliangyc@mail.scut.edu.cn (Y.L.); zwhu2016@163.com (Z.H.); yongcao@scut.edu.cn (Y.C.)

**Keywords:** photocatalysts, polymer blends, hydrogen evolution

## Abstract

We prepared a series of one-dimensional conjugated-material-based nanofibers with different morphologies and donor/acceptor (D/A) compositions by electrospinning for efficient photocatalytic hydrogen evolution. It was found that homogeneous D/A heterojunction nanofibers can be obtained by electrospinning, and the donor/acceptor ratio can be easily controlled. Compared with the single-component-based nanofibers, the D/A-based nanofibers showed a 34-fold increase in photocatalytic efficiency, attributed to the enhanced exciton dissociation in the nanofibrillar body. In addition, the photocatalytic activity of these nanofibers can be easily optimized by modulating the diameter. The results show that the diameter of the nanofibers can be conveniently controlled by the electrospinning feed rate, and the photocatalytic effect increases with decreasing fiber diameter. Consequently, the nanofibers with the smallest diameter exhibit the most efficient photocatalytic hydrogen evolution, with the highest release rate of 24.38 mmol/(gh). This work provides preliminary evidence of the advantages of the electrospinning strategy in the construction of D/A nanofibers with controlled morphology and donor/acceptor composition, enabling efficient hydrogen evolution.

## 1. Introduction

Hydrogen is a new energy source with extremely high potential for replacing fossil fuels because of its high energy density, low pollution, and low environmental impact [1,2,3]. Hydrogen can be produced by the photocatalytic splitting of water without any pollution. Therefore, developing highly efficient and cost-effective photocatalysts for the application of converting solar energy into storable hydrogen is desirable [4,5]. 

Organic semiconductor materials can be easily modified in terms of their electronic properties; their band gaps can be flexibly adjusted, and their wide absorption range and high charge carrier mobility have a wide range of applications in the field of photocatalytic hydrogen evolution [6,7,8,9,10,11]. For instance, graphitic carbon nitride derivatives [12,13,14], covalent organic frameworks [15,16,17,18], microporous conjugated polymers [19,20], and hydrophilic conjugated materials [21] have been explored for photocatalytic applications. However, as the exciton binding energy is too high (>0.3 eV), organic photocatalysts have severe charge recombination problems, resulting in a short exciton diffusion length (5–20 nm) [22,23,24]. Combining donor and acceptor materials to establish a D/A heterojunction structure can efficiently broaden the visible-light absorption range, reduce the compounding of photogenerated carriers, and achieve effective separation of excitons and a longer photogenerated carrier lifetime, which is well known in the OPV field [25]. This strategy also shows greater potential in photocatalytic hydrogen evolution [26,27,28]. In addition, previous research has proposed that organic photocatalysts with high water dispersion/solubility can shorten the paths of photoinduced excitons emigrating to the solid–liquid interface and enable high mobility/conductivity to reduce exciton complexation within polymers [29,30,31,32,33,34]. Nevertheless, the hydrophobic backbone and alkyl side chains of conjugated polymers prevent them from dispersing well in water [35,36]. However, there are significant challenges in controlling the composition and morphology of heterojunctions in the aqueous phase, and related investigations in this field have rarely been reported.

Electrospinning is a reliable method for constructing one-dimensional nanomaterials benefiting from the advantages of adjustable nanoscale size, controllable composition and morphology, persistent process, high specific surface area, good mechanical flexibility/strength, simple processes, and low production costs for large-scale applications [37,38,39,40,41,42,43,44]. These merits endow electrospun nanofibers with great potential for applications in the conversion of solar energy to restorable chemical energy. Recently, the hydrogen production efficiency of inorganic electrospun nanofibers has been evaluated, suggesting the promising potential of electrospun nanofibers in clean energy production [45,46,47,48,49]. Similarly, organic conjugated nanofibers offer the advantages of high specific surface area and controlled composition and morphology, and allow simply building heterojunction structures [50]; however, organic conjugated nanofibers have rarely been reported in photocatalytic hydrogen evolution research 

**Figure 1:**
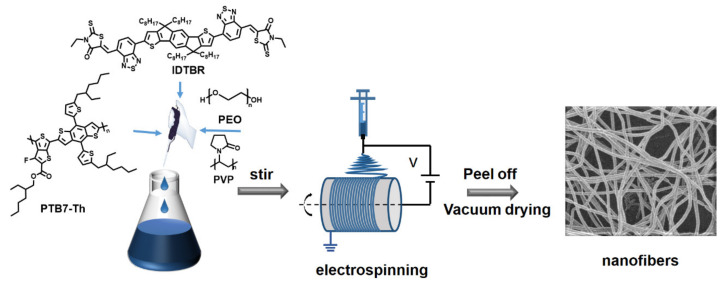
Schematic diagram of electrospinning setup.

Herein, we demonstrate a series of one-dimensional electrospun organic donor/acceptor (D/A) nanofibers, realizing controllable composition and morphology, as well as highly efficient photocatalytic evolution. Conjugated polymer PTB7−Th and nonfullerene acceptor EH−IDTBR (Figure 1) were separately selected as donor and acceptor to construct a heterojunction blend, which was then blended with hydrophilic polymers polyvinylpyrrolidone (PVP) and polyethylene oxide (PEO). Electrospinning was employed to fabricate the conjugated polymer nanofibers. The D/A heterojunction-based nanofibers exhibited a 34-fold increase in photocatalytic efficiency in comparison to the single-component fibers, attributed to the enhanced exciton dissociation in the nanofibrillar body of the former. Additionally, nanofibers with controllable diameter were realized by adjusting the solution concentration, spinning voltage, propulsion rate, temperature, and humidity of the electrospinning process. We revealed that both the diameter and component ratio of nanofibers can largely determine their photocatalytic activity. Therefore, finer nanofibers could achieve a large improvement in hydrogen evolution rates over thicker nanofibers. Moreover, polymer nanofibers containing PEO showed a 181% enhancement in the hydrogen evolution rate compared with that with PVP. Our investigation provides simple and efficient strategies to improve the photocatalytic activity of organic conjugated polymers, and these strategies are universal for other photocatalytic systems.

## 2. Materials and Methods

### 2.1. Materials and Solution Preparation

Semiconductor donor polymer poly([2,6′-4,8-di(5-ethylhexylthienyl) benzo [1,2-b;3,3-b]dithiophene] 3-fluoro-2[(2-ethylhexyl)car- bonyl]thieno [3,4-b]thiophenediyl (PTB7–Th, M_w_ = 72 kDa), nonfullerene acceptor 4-thiazolidinone, 5,5′-[[4,4,9,9-tetrakis(2-ethylhexyl)-4,9-dihydro-s-indaceno [1,2-b:5,6-b’]dithiophene-2,7-diyl]bis(2,1,3-benzothiadiazole-7,4-diylmethylidyne)]bis [3-ethyl-2-thioxo-(EH−IDTBR), Chloroplatinic acid (H_2_PtCl_4_), and ascorbic acid (AA) were purchased from Sigma-Aldrich Trading Co., Ltd. (Shanghai, China). Polyethylene oxide (PEO) and polyvinylpyrrolidone (PVP) were purchased from Aladdin. All chemical reagents were used as received, and all experiments and measurements were performed at room temperature unless specified.

Firstly, a solution of PTB7−Th/EH−IDTBR (mPTB7−Th/mEH−IDTBR = 3/7, 5/5, and 7/3, weight ratios) was prepared at a mass concentration of 20 mg/mL by a mixed solvent of CHCl_3_ and chlorobenzene (9:1 in volume) with vigorous stirring at 50 °C for 4 h. Then. PEO or PVP chloroform solution was prepared at a mass concentration of 20 mg/mL, mixed with the above solution in equal volumes, and stirred magnetically for 2–4 h to obtain a homogeneous electrospinning precursor solution.

The colloidal solution was then filtered through a 0.45 μm syringe filter before use. Concentrations of solutions for electrospinning were calibrated by taking the solvent evaporation during the fabrication process into account. All samples were prepared using the same method unless stated otherwise.

### 2.2. Electrospinning Experiments

**Figure 2:**
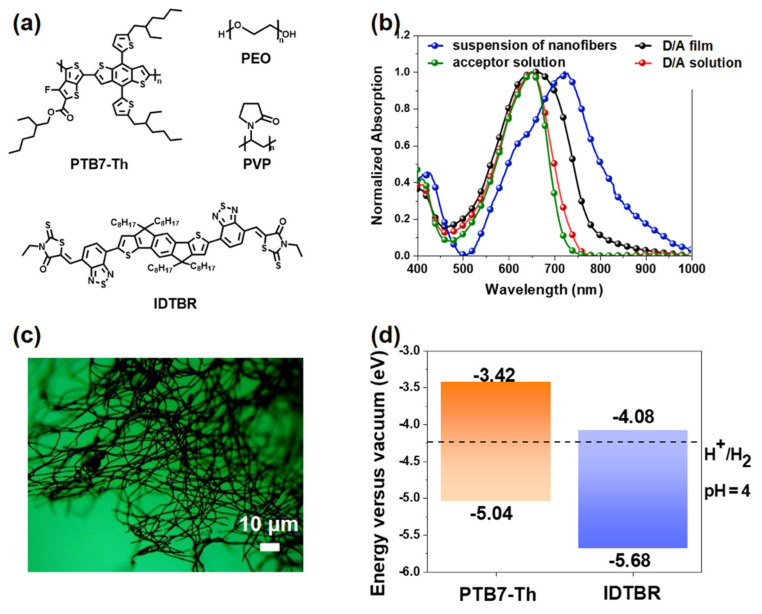
(**a**) Chemical structures of PTB7−Th, EH−IDTBR, PVP, and PEO. (**b**) UV-vis absorption spectra of different forms. (**c**) Highest occupied and lowest unoccupied molecular orbital energy levels of PTB7−Th and EH−IDTBR, as well as the proton reduction potential (H^+^/H_2_), water oxidation potential (O_2_/H_2_O), and calculated potential of the two-hole oxidation of ascorbic acid to dehydroascorbic acid in solution (DHA/AA) at pH 4 (experimentally measured pH of 0.2 mol L^−1^ ascorbic acid). (**d**) Nanofibers prepared at a feed rate of 0.15 mm/min. D/A ratio is 3:7.

The schematic fabrication process of the D/A heterojunction-based electrospun nanofibers and the resulting morphology are shown in Figure 1. Electrospinning experiments were carried out by using a 2.5 mL syringe equipped with a 21-gauge stainless steel needle with a diameter of ~0.5 mm as the anode for electrospinning, a syringe pump for controlled delivery of polymer solutions, a metal collector with a distance of 15–20 cm from the needle tip, and a high-voltage power supply. The mixture solutions composed of a donor polymer PTB7−Th and the nonfullerene acceptor EH−IDTBR (Figure 2a) were transferred into the syringe and injected through the needle at a constant rate of 0.08–0.20 mm/min in the air at room temperature and 40% relative humidity. A voltage bias of 6–10 kV was applied between the syringe needle and the collector. The surface shape of the charged droplets was successfully kept by regulating the magnitude of the voltage at the needle and the solvent system to obtain the so-called Taylor cone [51]. Then, the charged liquid jet was submitted to a spiraling motion when accelerated towards the collector. Meanwhile, the solvent evaporated rapidly, forming solid fibers, and 80-mesh stainless wire mesh was employed as the fiber deposition target.

### 2.3. Characterization

The morphologies of the conjugated electrospun nanofibers were inspected by optical microscopy (Olympus MX51, Tokyo, Japan) and field-e mission scanning electron microscopy (SEM; Hitachi S4800,Tokyo, Japan) at an accelerating voltage of 5 kV. The chemical analysis of the electrospun nanofibers was performed by transmission electron microscopy (TEM; JEM-2100F, Tokyo, Japan) with an accelerating voltage of 200 kV. The UV-visible absorption spectra of the aqueous dispersion of the electrospun nanofibers were recorded on an HP 8453 spectrophotometer (Palo Alto, CA, USA). For comparison, the film obtained by spin-coating an equal molar ratio of the EH−IDTBR and PTB7–Th chloroform solutions onto the quartz plate was also subjected to record its UV-visible absorption spectra. The photoluminescence spectra of conjugated polymers and their blends were recorded on a JobinYvon Fluorolog-3 spectrophotometer (Paris, France). Transmission FT-IR spectra were recorded on a Nicolet IS50 (Waltham, MA, USA) at room temperature, and samples were prepared by dropping the nanofiber aqueous dispersion on the pressed KBr pellets.

### 2.4. Photocatalytic Processes

The hydrogen evolution measurements were performed on a Labsolar-IIIAG photocatalytic system (PerfectLight, Beijing, China) equipped with a 50 mL reactor. For the photocatalytic experiment using AA as the sacrificial agent, the electrospun nanofibers (4 mg) were dispersed into a mixture of 50 mL of AA solution (0.2 M, pH = 4.0 adjusted by NaOH aq) with ultrasonic for 30 min. The total concentration of the electrospun nanofibers for photocatalytic measurement in the reaction solution was 80 µg/mL. Pt cocatalysts (3 wt% of the electrospun nanofibers) were prepared by dissolving H_2_PtCl_4_ aqueous solution into the reactor and irradiating for 1 h to enable the formation of Pt nanoparticles cocatalysts before testing. The reaction was sealed with a quartz septum and held under vacuum for 30 min to remove the dissolved oxygen before testing. The photocatalytic reactions were illuminated with a solar simulator (300 W Xe light source, λ > 300 nm). The luminous power reaching the surface of the reaction solution was calibrated to be 80 mW/cm^2^ by a power meter. The produced gas was analyzed by a GC7900 gas chromatograph (GC7900II, Shanghai, China using argon as carrier gas). Hydrogen was detected with a thermal conductivity detector, referencing standard gas with a known concentration of hydrogen. The sensor was standardized by injecting different volumes of hydrogen at the experimental condition and was polarized at +36 mV until reaching a stable value before every measurement.

## 3. Results and Discussion

In the present study, we prepared two separate sets of samples. Firstly, a set of nanofiber heterojunctions with different D/A ratios was obtained by adjusting the donor/acceptor ratio in the solution. In this set of samples, we prepared similar fiber diameters to study the effect of different D/A ratios on photocatalysis. Secondly, we fixed the donor/acceptor ratio and varied the fiber diameter by controlling the feed rate of the solution, to study the effect of fiber diameter on photocatalytic hydrogen evolution.

### 3.1. Absorption Spectroscopy Properties and Morphology of Electrospun Nanofibers

The optical properties of these nanofibers were examined by UV–visible–NIR absorption spectroscopy, as shown in Figure 2b. For comparison. PTB7−Th/EH-IDTBR heterojunction materials in chloroform solution as a cast film and nanofiber suspension were characterized, respectively. Since PEO and PVP are nonluminescent materials without absorption and emission signals, the absorption band attributed to the D/A materials remained the same as that after blending with elastomers. The absorption spectra of the D/A materials in the thin film were similar to that in the solution, but a slightly broader spectrum in the range of 735 to 783 nm was observed in the former, implying the formation of larger aggregation and higher crystallinity in the system. In contrast, a distinct absorption peak at 729 nm appeared in the D/A fiber suspension. The significant redshift of the fiber suspension could probably be attributed to reduced π–π stacking between chains and enhanced interchain interaction in the semicrystalline polymer domains induced by the electrospinning process, hence leading to smaller band gaps. The results suggest that electrospinning can enable stronger aggregation of the compound and facilitate exciton dissociation and transport. Furthermore, Figure S1 (Supplementary Materials) shows the UV–visible absorption spectra of the nanofiber dispersions before and after photocatalysis, demonstrating that the absorption properties of the nanofibers are stable after photocatalysis.

The visual evidence of the morphology of the electrospun fibers was obtained by optical microscopy, as shown in Figure 2c and Figure S2. Interestingly, electrospun nanofibers possess a uniform distribution and favorable nonwoven fabric shape, demonstrating that it is feasible to prepare nanofibers by electrospinning technology.

We selected the donor polymer PTB7−Th and the nonfullerene acceptor EH−IDTBR as sources to explore the potential of conjugated-polymer-based electrospinning in photocatalytic hydrogen evolution. According to previous reports, this D/A combination causes strong visible-light absorption, type II energy level offset [52,53], and finer interfacial morphology. Thus, efficient charge separation at the PTB7−Th/EH−IDTBR heterojunction was driven, and photoexcited electrons were generated, which could sufficiently reduce the EH−IDTBR lowest unoccupied molecular orbital (LUMO) energy levels to improve the hydrogen evolution rate (HER) with the aid of a Pt cocatalyst, as well as oxidizing holes in the PTB7−Th highest occupied molecular orbital to oxidize ascorbic acid (Figure 2d).

The PL spectra of the nanofiber suspension were obtained by exciting the sample, similar to that prepared for the hydrogen evolution reaction with a 630 nm laser. As shown in Figure S3, the nanofiber suspension exhibited a distinct fluorescence peak in the range of 700 to 850 nm due to the transition of the material from the excited state to the ground state.

**Figure 3:**
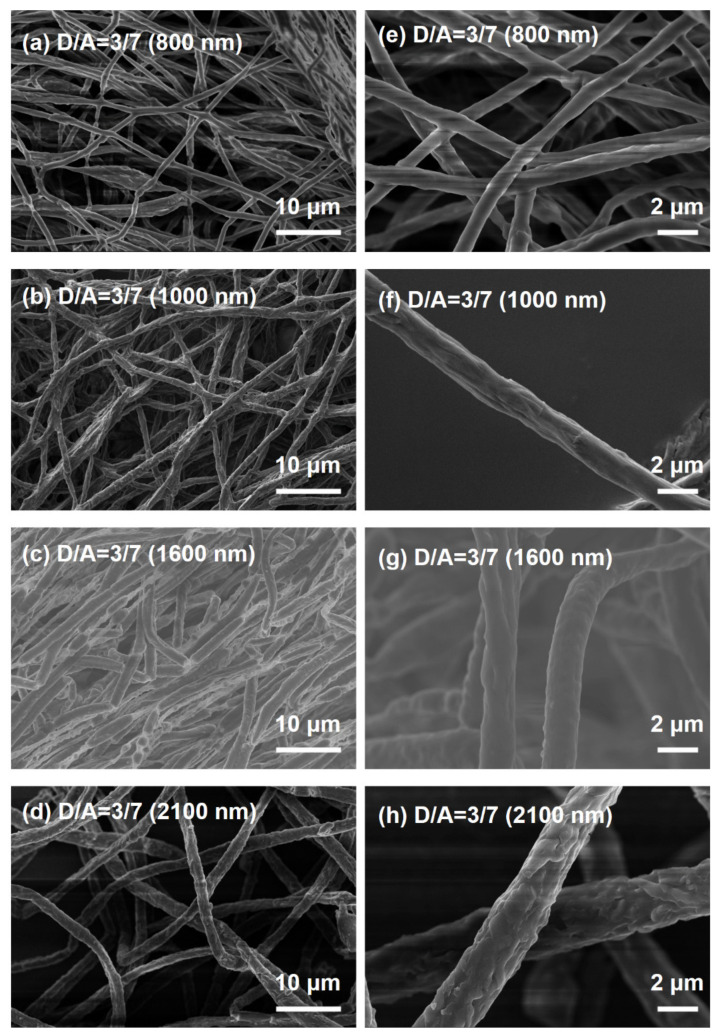
(**a**–**d**) SEM of fibers with different average diameters under low magnifications, where the donor/acceptor = 3/7 within the fibers. (**e**–**h**) Corresponding enlarged SEM images of (**a**–**d**).

In order to obtain a clearer morphology of the D/A electrospun nanofiber obtained with different feed rates, scanning electron microscopy (SEM) was employed, as shown in Figure 3. It can be observed that the average diameters of the fibers varied for different samples. Figure 3a–d shows the SEM of fibers with different average diameters under low magnifications, where the donor/acceptor = 3/7 within the fibers. Figure 3e–h shows corresponding enlarged SEM images of Figure 3a–d. A smooth surface structure of D/A electrospun nanofibers without bead formation was observed because both chlorobenzene (CB) (a good solvent for PTB7−Th and IDTBr with high boiling points) and chloroform (TCM) (a good solvent for PEO and IDTBr) were used as the mixed solvent (CB/ TCM = 1/9, *v*/*v*) for electrospinning. In addition, the diameter of the nanofibers could be easily tuned by controlling the electrospinning feed rate. For example, the average diameter of the D/A electrospun fibers significantly decreased from 2.1 μm to 0.8 μm by declining the solution feed rate from 0.20 to 0.08 mm/min. In addition, no typical signs of IDTBr or PTB7−Th aggregations could be observed in the fibers in our study.

Although all D/A solutions were electrospun successfully, the relationship between the dielectric constant of the solvent, which is a measure of the polarity of the solvent, and the average diameter of the fibers is still worth considering. It is proved that the solvent with a high dielectric constant has a higher net charge density in the solution. Therefore, as the charges carried by the jet increased, higher elongation forces were imposed on the jet under the electrical field, resulting in smaller beads and thinner fiber diameters [54,55]. However, the dielectric constant is 4.8 for chloroform and 5.6 for chlorobenzene at 25 °C. Such a low dielectric constant means that the solution has a poor ability to carry charges, and the solution is subjected to fewer elongation forces in the electrical field, which is unfavorable for electrospinning [56,57]. Based on the reasons mentioned above, the diameters of the nanofibers obtained in our study are not particularly satisfactory.

**Figure 4:**
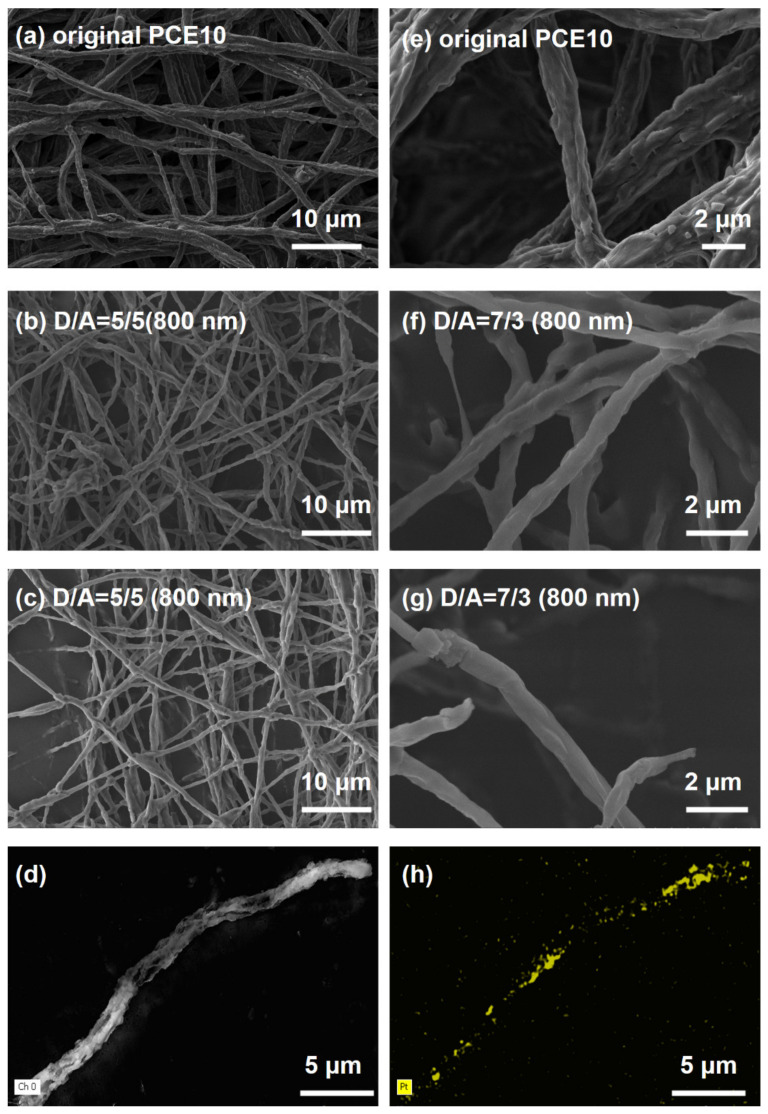
(**a**–**c**) SEM of fibers with different donor/acceptor ratio under low magnifications, where the average diameter of the fibers is about 800 nm. (**e**–**g**) Corresponding enlarged SEM images of (**a**–**c**). (**d**) SEM image of fiber with an average diameter of about 800 nm and donor/acceptor = 3/7 with Pt cocatalyst, and (**h**) corresponding elemental maps of Pt in (**c**).

In addition, to investigate the influence of composition on the hydrogen evolution, nanofibers with different D/A ratios and different matrixes were prepared by electrospinning, and the diameter was fixed at 800 nm by controlling the feed rate. The SEM images of electrospun fibers as stated above are shown in Figure 4 and Figure S4. The morphological characteristics of these electrospun nanofibers remain highly consistent with previously prepared nanofibers, demonstrating that the control of nanofiber diameter by feed rate is universal.

Meanwhile, at this point, the successful in situ reduction of the Pt cocatalyst on nanofibers was confirmed by characterizing the distribution of each element in the nanofibers via SEM mapping (Figure 4d,h). The corresponding EDS pattern of nanofibers is shown in Figure S5. Additionally, PTB7−Th could no longer fully encapsulate the EH−IDTBR nucleus, allowing more efficient extraction of electrons from the EH−IDTBR and holes from PTB7−Th, thus resulting in a maximum HER rate of 70%.

### 3.2. Photocatalytic Hydrogen Evolution

In an effort to investigate the effect of nanofiber diameter on the hydrogen evolution of these conjugated polymers-based electrospun nanofibers, a series of one-dimensional electrospun nanofibers with diameters of approximately 0.8–2.1 μm were dispersed into the photocatalytic reactor with a controlled mass of 4 mg for each test. Meanwhile, platinum was in situ photodeposited on the composite nanofiber surfaces during H_2_ evolution measurements.

**Figure 5:**
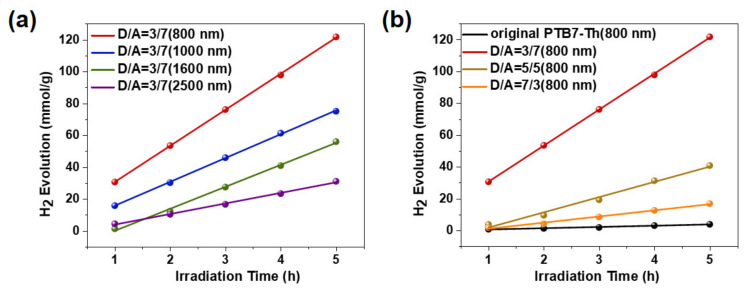
H_2_ evolution versus irradiation time of samples with (**a**) different diameters of nanofibers and (**b**) different compositions.

Nanofibers with different diameters and PEO matrixes were first tested, and the amount of generated H_2_ versus time is presented in Figure 5a. For the nanofibers with diameters of 0.8 μm, 1.0 μm, 1.6 μm, and 2.1 μm, 1218.9, 559.1, 308.8, and 35.5 μmol of H_2_ were produced in 5 h, respectively. These nanofibers achieved satisfactory levels of hydrogen evolution efficiency, especially the 0.8 μm nanofibers, which achieved 24.38 mmol/(gh). In addition, it could be found that the performances of the catalysts were significantly influenced by the diameters of the nanofibers. Thicker nanofibers displayed reduced hydrogen evolution efficiency in the experiment because the surface area of the thick nanofiber was smaller, and the exciton migration path was longer. In comparison, the smallest diameter of the D/A nanofibers fabricated in this experiment was 800 nm, which is not comparable with the distance of the electron transfer and the exciton diffusion length. This demonstrates that high photocatalytic hydrogen evolution efficiency may originate from the fact that part of the active subchains is effectively stretched and oriented along the fiber axis during electrospinning, adopting a more extended chain conformation, accompanied by an increase in the associated conjugation length and the arrangement that supports interchain interactions with nearby subchains.

In an attempt to determine the effect of different D/A composition mass ratios on the efficiency of the photocatalytic hydrogen evolution of electrospun nanofibers, fibers using a uniform PEO matrix containing D/A mass ratios of 3:7, 5:5, 7:3, and 10:0 were prepared, and their corresponding photocatalytic hydrogen production was tested under the same conditions as mentioned above, as shown in Figure 5b. Compared with the photocatalytic hydrogen evolution generated by the single-component-based nanofiber, which exhibited a limited performance of 0.7 mmol/(gh), a significant improvement was obtained in the D/A-based electrospun nanofibers, attributed to the enhanced charge generation and exciton dissociation in the composite nanofibers induced by the internal D/A heterojunction [58,59]. Since Pt particles normally deposit on the EH−IDTBR core, increasing the EH−IDTBR fraction could improve the exposure probability of the EH−IDTBR core on the surface of nanofibers, thus contributing to the enhancement of electron extraction from the nanofibers to a certain extent. The highest photocatalytic hydrogen production efficiency is obtained when the D/A mass ratio is 3/7 in this study.

**Figure 6:**
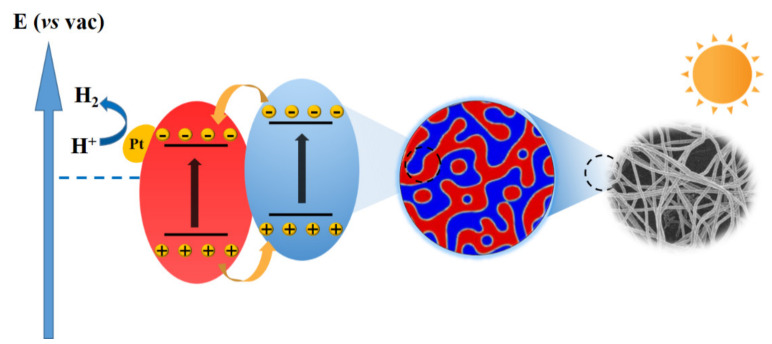
Schematic illustration for the possible photocatalytic mechanism of the H_2_ generation in nanofibers under xenon lamp irradiation.

Given the low molecular weight, chain rigidity, and limited solubility of conjugated polymers, especially small-molecule organic semiconductors with poor molecular entanglement, preparation of conjugated polymers-based nanofibers by electrospinning normally requires the assistance of high-molecular-weight flexible chain-insulating polymers. PEO and PVP, two widely used flexible insulating polymers, were employed to blend with the conjugated polymers for electrospinning, and the effect of different matrixes on the photocatalytic hydrogen evolution performance of nanofiber was investigated (Figure S6). Although the inclusion of a water-soluble component in the electrospun nanofibers could somewhat cause their degradation in water, the performance to induce photocatalytic hydrogen evolution is not affected. Notably, the hydrogen evolution efficiency obtained by the PEO matrix nanofibers is 2.81 times higher than that based on the PVP matrix nanofibers. The difference in HER caused by the two different matrixes might originate from their different particle-phase separation sizes or porous structure on the fiber surfaces [60,61,62]. A possible photocatalytic mechanism of the H_2_ generation in nanofibers under xenon lamp irradiation is shown in Figure 6.

### 3.3. Stability

In addition, the photocatalytic stability of the nanofiber suspension was evaluated via a continuous photocatalytic reaction under irradiation for 20 h. The amount of hydrogen generated as a function of time is shown in Figure S7a. It can be seen that the yield of hydrogen climbed steadily with the irradiation time. Furthermore, the average HERs remained at a high level in the first three cycles of the photocatalytic experiments: 23.42 mmol g^−1^ h^−1^ for Run 1, 18.55 mmol g^−1^ h^−1^ for Run 2, and 19.88 mmol g^−1^ h^−1^ for Run 3, which is probably attributed to the high photocatalytic activity, as well as the dispersion of nanofibers in the first few cycles. The prolongation of irradiation resulted in a decrease in HER to 6.36 mmol g^−1^ h^−1^ for Run 4. Nanofibers before and after photocatalysis were analyzed by Fourier-transform infrared spectroscopy (FT-IR), as shown in Figure S7b. The alkyl C–H functionalities at the wavenumber of 2922 cm^−1^ were attributed to PEO contained in the electrospun fibers. After the photocatalytic reaction, the stretching bands at 2922 cm^−1^ disappeared, indicating that PEO was removed from the nanofibers during the hydrogen evolution process. However, few strong peaks presented at 1705 cm^−1^, 1650 cm^−1^, 1320 cm^−1^, and 1110 cm^−1^, which corresponded to the C=O, C=C, C–N, and C–F stretching, respectively, suggesting that D/A nanofibers have long-term photostability.

Meanwhile, in our study, we prepared nanofibers with a diameter of about 800 nm and a donor/acceptor/PEO mass ratio of 3/7/10, and Figure S8 demonstrates the morphology of the fibers after the reaction. It can be seen that the fibers were able to maintain their morphology after the reaction, with a reduction in diameter and an increase in surface porosity, probably due to the loss of water-soluble PEO. At the same time, there was an increase in phase separation, caused by the phase separation of the donor and acceptor components of the fibers, and in situ generation of Pt nanoparticles on the fibers could be observed.

## 4. Conclusions

In summary, we successfully prepared a series of one-dimensional donor/acceptor (D/A) heterojunction-based conjugated polymer nanofibers using electrospinning technology and achieved efficient photocatalytic hydrogen evolution. The relationship between nanofiber diameter and photocatalytic hydrogen production efficiency was explored, and efficiencies of up to 24 mmol/(gh) in the visible range were obtained. The D/A composition of the conjugated polymer nanofibers was optimized to obtain high photocatalytic hydrogen production. In addition, by blending different hydrophilic polymers with the D/A conjugated polymers for electrospinning, an optimal matrix was selected to obtain efficient photocatalytic hydrogen evolution. Overall, this work proves that electrospinning is an effective strategy to enhance the photocatalytic activity of conjugated polymers, thus expanding the application potential of conjugated polymer-based nanofibers.

## Data Availability

Not applicable.

## Supplementary Materials

The following supporting information can be downloaded at: https://www.mdpi.com/article/10.3390/nano12091535/s1, Figure S1: UV–vis absorption spectra of nanofiber dispersions before and after photocatalysis. Figure S2: Optical microscope picture of nanofibers prepared at a feed rate of (**a**) 0.08 mm/min (**b**) 0.10 mm/min, and (**c**) 0.20 mm/min; (**d**) 80-mesh stainless steel wire mesh and the fibers prepared at a feed rate of 0.10 mm/min on it. Figure S3: Photoluminescence spectra of original PTB7−Th and nanofibers with D/A = 3/7 in water suspension. Figure S4: Typical SEM images of (**a**) the calcined products of nanofibers prepared from PVP as support material under low magnifications and (**b**) representative SEM images under high magnifications. Figure S5: EDS pattern of D/A = 3/7 nanofibers (800 nm). Figure S6: H_2_ evolution versus irradiation time of samples with different support materials. Figure S7: (**a**) Hydrogen evolution of D/A nanofibers over long-time illumination. Average HERs of 23.42 mmol/(gh) for Run 1, 18.55 mmol/(gh) for Run 2, and 19.88 mmol/(gh) for Run 3. (**b**) Transmission FT-IR spectra of nanofibers before and after photocatalysis. Figure S8: SEM images of the fibers with an average diameter of about 800 nm and donor/acceptor = 3/7 after reaction.
