# Supplementary material for: Electrospun Donor/Acceptor Nanofibers for Efficient Photocatalytic Hydrogen Evolution
Source: Nanomaterials (Basel). 2022 May 2;12(9):1535. doi: 10.3390/nano12091535 (PMC9101664; doi:10.3390/nano12091535)
Supplement: Supplementary file 1 [file nanomaterials-12-01535-s001.zip › nanomaterials-1689849-supplementary.pdf]

nanomaterials

MDPI

*Supplementary Materials*

# Electrospun Donor/Acceptor Nanofibers for Efficient Photo-catalytic Hydrogen Evolution

**Xiaoyu Lin, Yuanying Liang, Zhicheng Hu *, Xi Zhang, Youcai Liang, Zhengwei Hu, Fei Huang * and Yong Cao**

State Key Laboratory of Luminescent Materials and Devices, Institute of Polymer Optoelectronic Materials and Devices, South China University of Technology, Guangzhou 510640, China;
201920117734@mail.scut.edu.cn (X.L.); liangyuanying1@hotmail.com (Y.L.);
201710102620@mail.scut.edu.cn (X.Z.); msliangyc@mail.scut.edu.cn (Y.L.);
zwhu2016@163.com (Z.H.); yongcao@scut.edu.cn (Y.C.)

* Correspondence: scut_hzc@126.com (Z.H.); msfhuang@scut.edu.cn (F.H.)

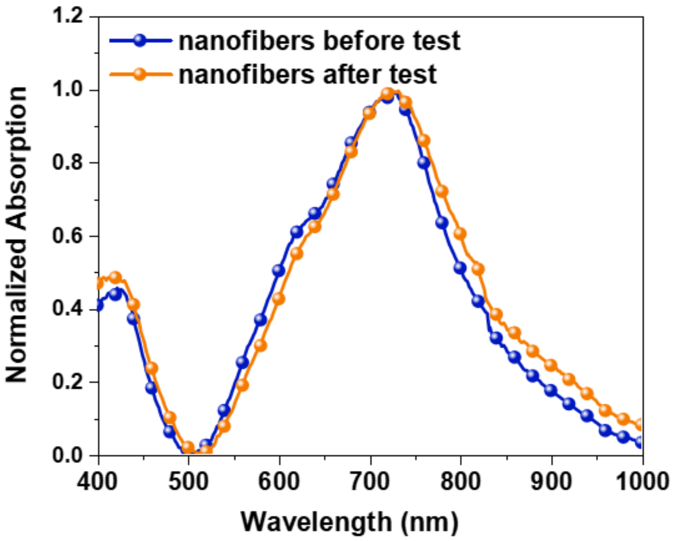


**Figure S1.** UV-Vis absorption spectra of nanofibers dispersions before and after photocatalysis.

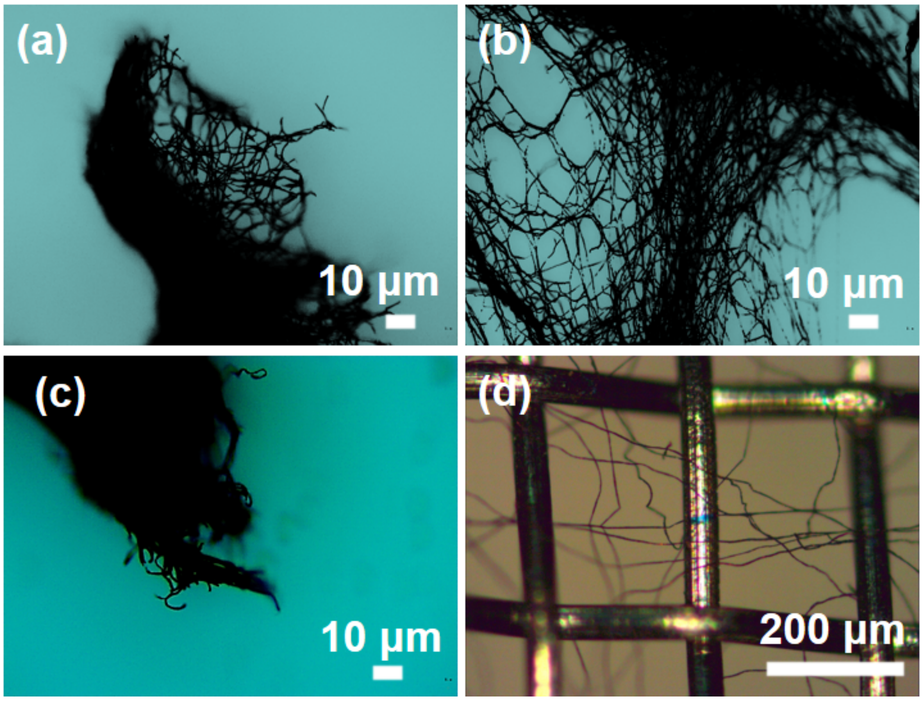


**Figure S2.** Optical microscope picture of nanofibers prepared at a feed rate of (**a**) 0.08 mm/min (**b**) 0.10 mm/min, and (**c**) 0.20 mm/min, (**d**) 80 mesh stainless steel wire mesh and the fibers prepared at a feed rate of 0.10 mm/min on it.

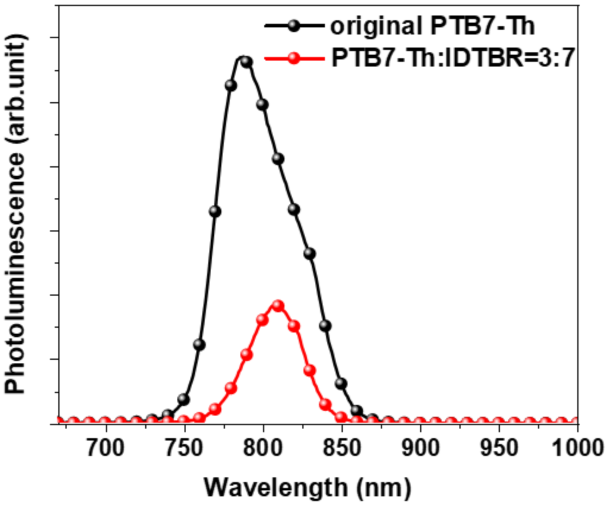


**Figure S3.** Photoluminescence spectra of original PTB7-Th and nanofibers with D/A=3/7 in water suspension.

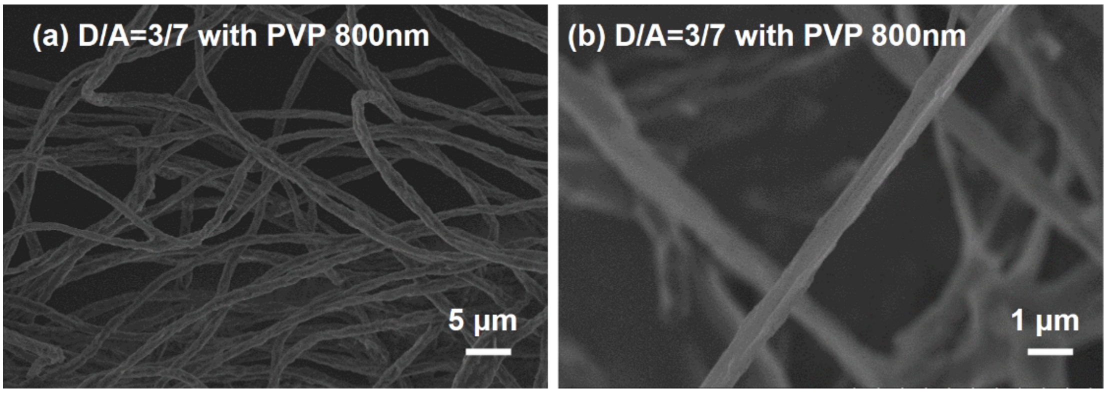


**Figure S4.** Typical SEM images of (**a**) the calcined products of nanofibers prepared from PVP as support material under low magnifications, and (**b**) representative SEM images under high magnifications.

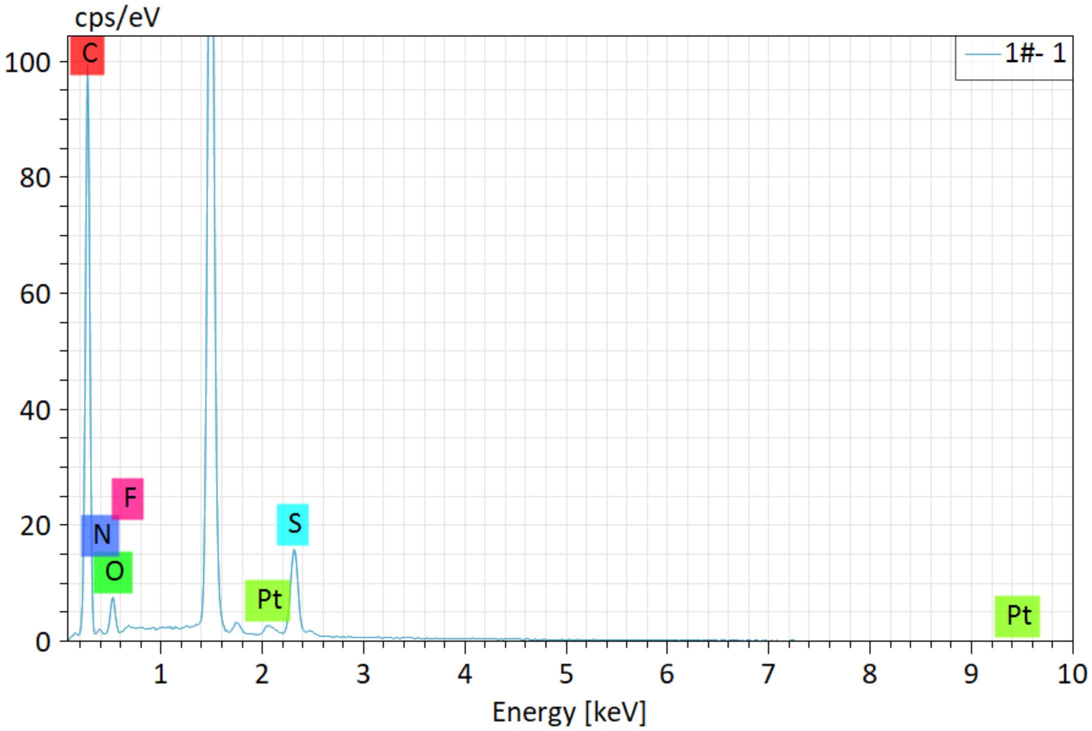


**Figure S5.** EDS pattern of D/A=3/7 nanofibers (800 nm).

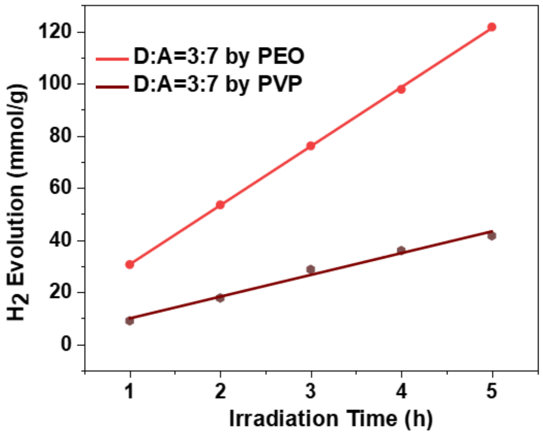


**Figure S6.** $H_2$ evolution versus irradiation time of samples with different support materials.

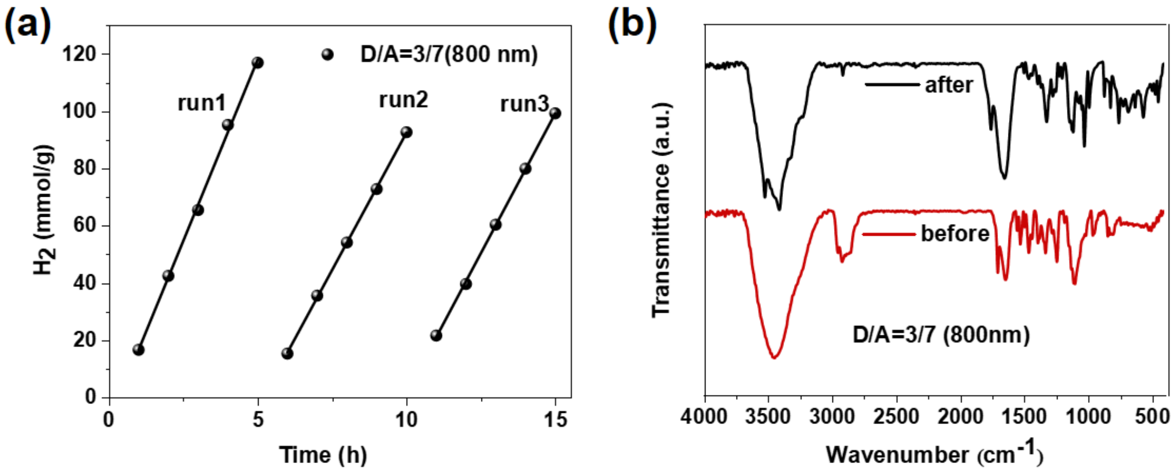


**Figure S7.** (**a**) Hydrogen evolution of D/A nanofibers with over long-time illumination. Average HERs of 23.42 mmol/(gh) for Run 1, 18.55 mmol/(gh) for Run 2, 19.88 mmol/(gh) for Run 3. (**b**)Transmission FT-IR spectra of nanofibers before and after photocatalysis.

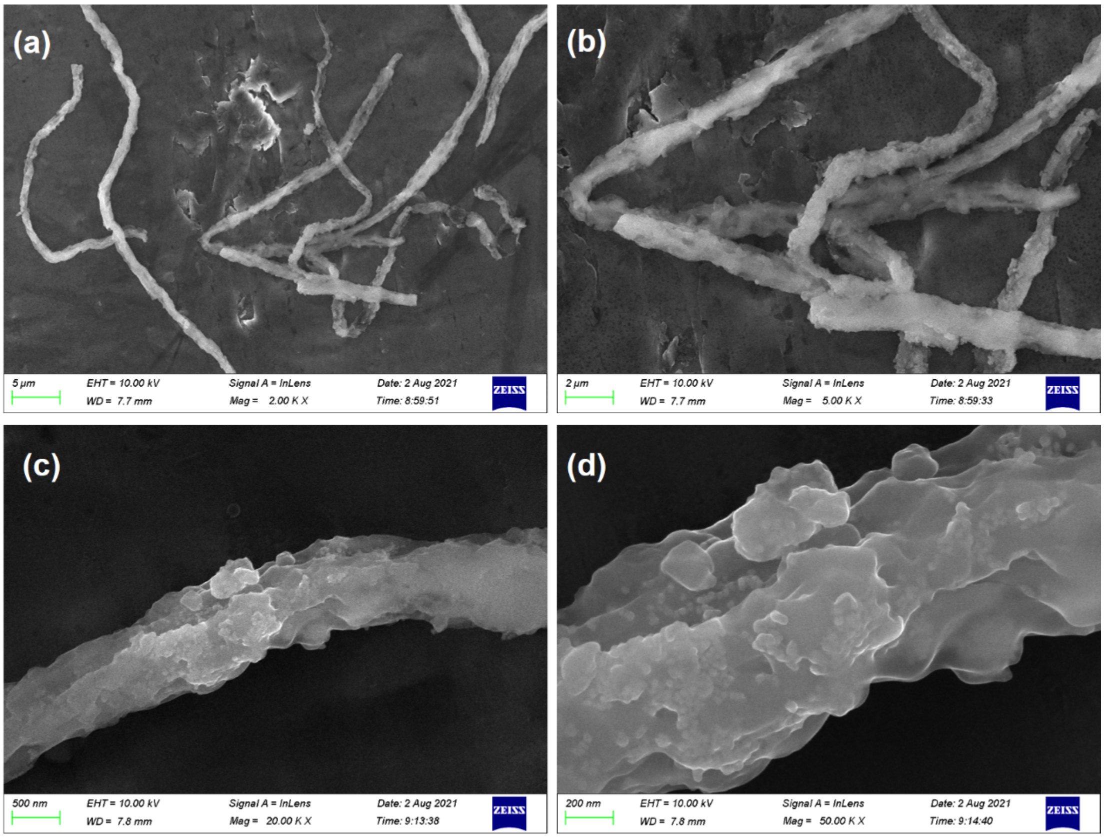


**Figure S8.** SEM images of the fibers with an average diameter of about 800 nm and donor/acceptor = 3/7 after reaction.
